# Oleanolic acid alleviates ovarian cancer by regulating the miR-122/PDK4 axis to induce autophagy and inhibit glycolysis *in vivo* and *in vitro*

**DOI:** 10.3389/fonc.2025.1670758

**Published:** 2025-09-03

**Authors:** Zhen Zeng, Qing Lin, Jing Yu, Min Lin, Ningwei Zhao

**Affiliations:** ^1^ Department of Physiology and Pathophysiology, School of Basic Medical Sciences, Fujian Medical University, Fuzhou, China; ^2^ Laboratory of Clinical Applied Anatomy, School of Basic Medical Sciences, Fujian Medical University, Fuzhou, China; ^3^ China Exposomics Institute, Shanghai, China

**Keywords:** ovarian cancer, oleanolic acid, miR-122, PDK4, glycolysis, autophagy

## Abstract

**Background:**

Ovarian cancer (OC) is a gynecological tumor with a high incidence and poor prognosis. Oleanolic acid (OA) plays a crucial role in cancers with its anti-cancer function. The study aimed to identify the effects of OA on OC development *in vivo* and *in vitro*.

**Methods:**

The cell viability, migration, and invasion were analyzed by the CCK-8 approach and the Transwell assay. The glycolysis was evaluated by the glucose uptake rate, lactate content, and glycolysis-related protein expression. The autophagy was analyzed by determining autophagy-related protein expression. The tumor volume and weight were measured. The H&E and immunohistochemical staining were performed to determine pathological injuries and Ki67 expression of the tumor tissue. The levels of miR-122 and PDK4 were measured by qRT-PCR.

**Results:**

OA inhibited the cell viability, migration, invasion, and glycolysis, and induced the autophagy of OC cells in a dose-dependent manner. Moreover, miR-122 was down-regulated in OC cells and increased by OA. Knocking down miR-122 effectively reversed the effects of OA on OC cells. PDK4 was clarified as a miR-122 target. Moreover, OA suppressed tumor volume and weight and Ki67 expression but induced pathological injuries of in tumor tissue. *In vivo* and *in vitro*, the overexpression of PDK4 and miR-122 effectively abolished the effects of OA and the overexpression of PDK4 on OC cells and tumor tissue, respectively.

**Conclusions:**

In conclusion, OA induced autophagy and inhibited glycolysis to attenuate OC progression by regulating the miR-122/PDK4 axis, providing a theoretical basis for clinical treatment of OC with OA, and novel therapeutic targets of OC.

## Highlights

- Oleanolic acid inhibited OC cell development and OC growth *in vivo* and *in vitro*.
- Oleanolic acid increased miR-122 expression in OC, which targeted PDK4.
- Oleanolic acid induced autophagy and suppressed glycolysis of OC via regulating the miR-122/PDK4 axis.

## Introduction

Ovarian cancer (OC) is the third most common gynecologic tumor, but has the highest mortality rate (1). The morbidity of OC is unchanged or declining in developed countries, but increasing in developing countries, with the 5-year survival rate of OC being less than 45% (2). Early screening can significantly improve the survival rate; however, there are no reliable diagnostic markers that can definitively diagnose the initiation of OC (3). Most patients are diagnosed at an advanced stage and cannot be completely cured with current treatments, so recurrence is extremely common (4). Therefore, it is urgent to further elucidate the pathogenesis of OC, control its progress, and explore effective treatment.

Autophagy is one of the ways that maintains cellular homeostasis, mainly participating in the occurrence and development of tumors by degrading excess molecules to produce new substances (5). It has been proposed as a way of programmed cell death. It was found that the abnormal expression of autophagy-related genes and proteins in OC cells regulated the apoptosis resistance, gene stability, and metabolic disorders, thereby affecting the progression of OC (6). Additionally, because of the high metabolic activity of cancer cells, they obtain energy through glycolysis (7). Even though glycolysis is less productive than oxidative phosphorylation. However, under hypoxic conditions, glycolysis is still the main energy generation pathway (8). Moreover, autophagy is associated with glycolysis in cancers (9). Therefore, targeting autophagy and glycolysis may be an effective strategy for OC.

Despite the emergence of chemically synthesized drugs, natural products remain the basis and major resource for drug innovation. Hitherto, half of the drugs on the market are derived from natural products (10). Oleanolic acid (OA) is a pentacyclic triterpenoid compound that can be isolated from a variety of plants, especially those in the Oleaceae family (11, 12). It has a variety of pharmacological effects, including liver protection, anti-inflammatory, antiviral, antibacterial, antioxidant, and anticancer activity (13). The anti-tumor activity of OA has been identified in numerous cancers, such as non-small cell lung cancer (14), hepatocellular carcinoma (15), pancreatic cancer (16), and OC (17). OA inhibits OC cell proliferation, invasion, and epithelial-mesenchymal transition (17). One previous study affirmed that the OA derivative, K73-03, induced autophagy in pancreatic cancer by regulating the miR-421/SPINK1 pathway (18). In addition, another previous study demonstrated that OA inhibited the proliferation of gastric cancer cells by regulating metabolism to suppress glycolysis, as reflected in the decrease in the uptake and consumption of glucose, intracellular lactate levels, and extracellular acidification rate (19). However, whether OA affects the autophagy and glycolysis of OC is largely unknown.

Therefore, the present study aimed to investigate whether OA affected autophagy and glycolysis of OC by regulating the miR-122/pyruvate dehydrogenase kinase 4 (PDK4) axis *in vivo* and *in vitro*, providing a theoretical basis for clinical treatment of OC with OA, and novel therapeutic targets of OC.

## Materials and methods

### Cell culture

The OC cell line (SKOV-3 and CAOV3) and normal ovarian epithelial cell line (IOSE-80 cells), bought from American Type Culture Collection (Virginia, USA), were cultured in RPMI 1640 medium (Cat., 11875093, Gibco, Massachusetts, USA) supplemented with 10% fetal bovine serum (Cat., A5670701, Gibco, Massachusetts, USA) and 100 U/mL penicillin with 100 mg/mL streptomycin (Cat., 15070063, Gibco, Massachusetts, USA). All cells were maintained under 37 °C with 5% CO_2_ conditions.

### Cell transfection

The miR-122 inhibitor, miR-122 mimic, inhibitor NC, PDK4 overexpression vector, and empty vector were purchased from Genepharma (China). The SKOV-3 and CAOV3 cells were transfected with these transfectants using Lipofectamine 3000 (Invitrogen, USA) for 72 hours before the OA treatment.

### Cell experiment grouping

To investigate the effects of OA on SKOV-3 and CAOV3 cells, they were randomly divided into five groups, including the control, DMSO, OA (low), OA (middle), and OA (high) groups. SKOV-3 and CAOV3 cells in the control group were normally cultured. Apart from the former two groups, SKOV-3 and CAOV3 cells in the latter three groups were treated with OA (Cat., O5504, purity≥97%, Sigma-Aldrich, Missouri, USA) at low- (25 µg/mL), middle- (50 µg/mL), and high- (100 µg/mL) concentration for 12 h, which were dissolved in the dimethyl sulfoxide (DMSO, Lot, 472301; Sigma-Aldrich, Missouri, USA) and diluted by the culture medium (20). SKOV-3 and CAOV3 cells in the DMSO group were treated with the DMSO vehicle at the same concentration. To determine whether OA regulates miR-122 in SKOV-3 and CAOV3 cells, they were randomly divided into four groups, including DMSO, OA, OA + inhibitor NC, and OA + miR-122 inhibitor groups. SKOV-3 and CAOV3 cells in the DMSO group were treated with the DMSO vehicle at the same concentration. Apart from the DMSO group, SKOV-3 and CAOV3 cells in other groups were treated with 100 µg/mL OA for 24 h. In addition, SKOV-3 and CAOV3 cells in the latter two groups were additionally transfected with corresponding transfectants for 72 hours before the OA treatment. To determine whether OA regulates the miR-122/PDK4 axis in SKOV-3 and CAOV3 cells, they were randomly divided into five groups, including DMSO, OA, OA + vector, OA + PDK4, and OA + PDK4 + mimic groups. SKOV-3 and CAOV3 cells in the DMSO group were treated with the DMSO vehicle at the same concentration. Apart from the DMSO group, SKOV-3 and CAOV3 cells in other groups were treated with 100 µg/mL OA for 24 h. In addition, the empty vector, PDK4 overexpression vector, and PDK4 overexpression vector with miR-122 mimic were additionally transfected to SKOV-3 and CAOV3 cells in corresponding groups before the OA treatment, respectively.

### Cell counting kit-8 assay

After finishing the treatments as mentioned above, the 10 μL CCK-8 solution (Cat., CK04, Dojindo, Kyoto, Japan) was subsequently incubated with SKOV-3 and CAOV3 cells for 4 h. The absorbance was examined using a Multiskan™ FC microplate reader (Thermo Fisher Scientific, Massachusetts, USA).

### Transwell assay

The cell migration was evaluated using Transwell chambers without Matrigel, and the cell invasion was evaluated using Transwell chambers pre-coated with Matrigel. After SKOV-3 and CAOV3 cells were suspended in serum-free medium and added into the top chamber, the RPMI-1640 supplemented with 10% FBS (600 μL) was added into the bottom chamber. After incubation for 24 h, the chambers were taken out. The cells in the bottom chamber were fixed with 4% paraformaldehyde and stained with 0.1% crystal violet (Cat., C0121, Beyotime, Shanghai, China). The stained cells were imaged and counted under a Mateo TL microscope (Leica, Hessen, Germany).

### Glucose uptake and lactate production assays

The glucose uptake and lactate production of SKOV-3 and CAOV3 cells were detected using the glucose uptake assay kit (Cat., MAK542) and lactic acid assay kit (Cat., MAK329) according to the manufacturer’s protocol. The two kits were both purchased from Sigma-Aldrich (Missouri, USA).

### Dual luciferase reporter analysis

The binding sites between miR-122 and PDK4 were predicted using the TargetScan database, among which PDK4 was predicted as a possible target. To verify the targeting relationship between miR-122 and PDK4, the wild-type (WT) and mutant (MUT) sequences of PDK4 were cloned into pGL3 vectors (Promega, USA). SKOV-3 cells were co-transfected with WT-PDK4 or MUT-PDK4 together with miR-122 mimic or mimic NC using Lipofectamine 3000. After transfection of 72 h, the luciferase activity was detected using the dual-luciferase reporter assay system (Cat., E1910, Promega, Wisconsin, USA).

### Animals and establishing the xenograft model

A total of 24 female BALB/c nude mice (4 – 6 weeks, weighed 12 – 18 g; Charles River Laboratories, Beijing, China) were adaptively fed in an environment with a 12 h light-dark alternation and approximately 22°C and 50% humidity for two weeks. During the adoption period, all mice were free of food and water. According to the previous study, the 5×10^6^ SKOV-3 cells with or without transfection suspended in 200 μL PBS were subcutaneously injected into the left anterior armpit of mice for model establishment (21). The animal study procedures followed the “Guide for the Care and Use of Laboratory Animals, 8th Edition”, and were approved by the Ethics Committee of Fujian Medical University (Ethical No.: IACUC FJMU 2022-0893).

### Animal experiment grouping and tumor volume and weight assays

To explore the effects of OA on OC *in vivo*, based on the procedure of simple randomization, the mice were randomly divided into four groups (n = 6) until the tumors grew to 100 mm^3^, including the control, OA, OA + PDK4, and OA + PDK4 + mimic groups. The normally cultured SKOV-3 cells without transfection were subcutaneously injected into mice in the control and OA groups. The SKOV-3 cells transfected with the PDK4 overexpression vector and the PDK4 overexpression vector with miR-122 mimic were subcutaneously injected into mice in the latter two groups. In addition, apart from the control group, mice in the latter three groups were treated with OA (50 mg/kg, i.g.) for 28 days (22). The length and width of the tumor were measured every week to calculate its volume using the formula (1/2 × Length × Width^2^). After 28 days, all mice were anesthetized with pentobarbital sodium (50 mg/kg, i.p.). Then, they were sacrificed, and the tumor tissue was collected for photographing, weighing, and subsequent studies.

### H&E staining

After the fixed and embedded tumor tissue was cut into 3-μm sections, they were deparaffinized and rehydrated. Then, the sections were stained with hematoxylin for 5 min. After that, they were washed using tap water until the cell nuclei were blue. Finally, the sections were stained with eosin for 2 min until the cytoplasm was pink. The stained results were observed under the microscope.

### Immunohistochemical assay

After the fixed and embedded tumor tissue was cut into 4-μm sections, they were deparaffinized in xylene and rehydrated with ethanol. Next, the citrate buffer and normal goat serum were used for antigen retrieval and blocking. After that, the sections were incubated with anti-Ki-67 (1: 200, Cat., ab15580) at 4 °C overnight, and immersed in the goat anti-rabbit IgG H&L (HRP) antibody (1: 1000, Cat., ab6721) at 37 °C for 1 h. All antibodies were bought from Abcam (Cambridge, UK). Finally, after being stained with the DAB solution, the stained results were visualized under the microscope, and the positively stained rate of Ki-67 was analyzed using the Image J software according to the proportion of positively stained cell number and total cell number.

### Quantitative real-time PCR

The total RNA in SKOV-3 and CAOV3 cells was extracted using the TRIzol reagent (Cat., 15596026). Following RNA purity and concentration determination, based on the following reaction conditions, 25°C for 10 min and 0 cycle, 42°C for 15 min and 0 cycle, 85°C for 5 min and 1 cycle, and 4°C for 1 min and 1 cycle, the reverse transcription was conducted using the miRNA 1st Strand cDNA synthesis kit (by tailing A) (Cat., MR201-01) for miRNAs and the HiScript II 1st Strand cDNA synthesis kit (Cat., R211-01, Vazyme, Nanjing, China) for mRNAs. Based on the following reaction conditions, 95°C for 10 min and 0 cycle, 95°C for 0.25 min and 50 cycles, 60°C for 1 min and 50 cycles, 95°C for 0.25 min and 0 cycle, 60°C for 1 min and 0 cycle, and 95°C for 0.25 min and 0 cycle, the real-time PCR was performed using the miRNA Unimodal SYBR qPCR Master Mix (Cat., MQ102-01) for miRNAs and the ChamQ Universal SYBR qPCR Master Mix (Cat., Q711-02) for mRNAs. All kits were obtained from Vazyme (Nanjing, China). The relative levels of miR-122 and PDK4 mRNA were calculated using the 2^-ΔΔCt^ method. The primer sequences were listed in **Table 1**, among which U6 and GAPDH were the endogenous controls of miR-122 and PDK4.

**Table 1:** The primer for qRT-PCR.

Primers	Forward/Reverse	Sequence
miR-122	Forward	CGCGTGGAGTGTGACAATGG
	Reverse	AGTGCAGGGTCCGAGGTATT
PDK4	Forward	CAATGGCACAAGGAATCATAGA
	Reverse	TCATCAGCATCCGAGTAGAAAT
U6	Forward	CTCGCTTCGGCAGCACA
	Reverse	AACGCTTCACGAATTTGCGT
GAPDH	Forward	GAGTCAACGGATTTGGTCGT
	Reverse	TTGATTTTGGAGGGATCTC

### Western blotting

After the RIPA buffer was used to lyse SKOV-3 and CAOV3 cells as well as tumor tissue for 2 min, the protein concentration was examined using the BCA kit (Cat., P0012, Beyotime, Shanghai, China). The protein samples were loaded into the 10% SDS-PAGE and then transferred to PVDF membranes. Next, the membranes were incubated with the specific anti-p62 (1: 1000, Cat., ab109012), anti-Beclin-1 (1: 2000, Cat., ab207612), anti-glucose transporter type 1 (GLUT1, 1: 1000, Cat., ab115730), anti-hexokinase 2 (HK2, 1: 1000, Cat., ab209847), and anti-PDK4 (1: 1000, Cat., ab214938), and anti-GAPDH (1: 2500, Cat., ab9485) at 4 °C overnight and the goat anti-rabbit IgG H&L (HRP) antibody (1: 10000, Cat., ab6721) at 25 °C for 2 h. All antibodies were bought from Abcam (Cambridge, UK). After the bands were visualized using the ECL reagent (Cat., abs920, Absin, Shanghai, China) for 3 min, their grayscale value were analyzed with the help of the Image J software.

### Statistical analysis

Data acquired from three independent experiments were shown as mean ± standard deviation and presented in figures using the GraphPad Prism 7 software. All generated data were used for statistical analysis without exclusion. Differences between the two groups were analyzed using the Student’s *t*-test, and differences among multiple groups were analyzed using one-way or two-way analysis of variance followed by Tukey’s *post-hoc* test. According to a previous study, the statistical power was calculated using simulations and with a power formula (23). After calculating, the statistical power of all statistical data achieved 80%. The statistical effect sizes were analyzed using the fixed effect model and represented by a 95% confidence interval. *P* < 0.05 was considered statistically significant.

## Results

### OA suppressed cell viability, migration, and invasion of SKOV-3 and CAOV3 cells

**Figure 1:**
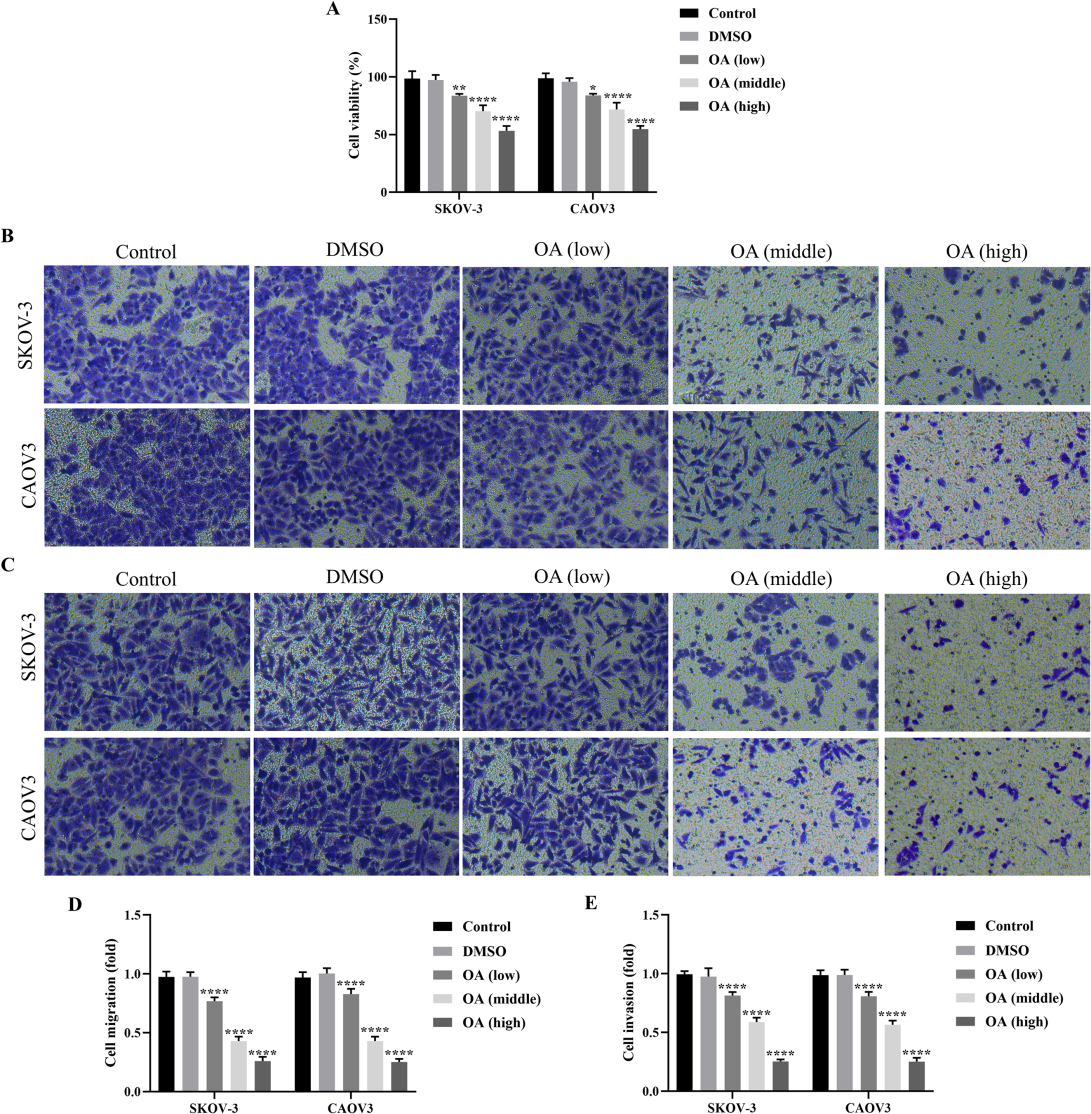
OA suppressed cellular viability, migration, and invasion of SKOV-3 and CAOV3 cells. **(A)** The cell viability of SKOV-3 and CAOV3 cells was assessed using the CCK-8 approach (n = 3). The representative images of the migrated **(B)** and invaded **(C)** SKOV-3 and CAOV3 cells were detected by the Transwell assay (magnification: 200 ×). The statistical analysis of the cell migration **(D)** and invasion **(E)** of SKOV-3 and CAOV3 cells (n = 5). OA, oleanolic acid. ^*^ *P*<0.05, ^**^ *P*<0.01, ^***^ *P*<0.001, and ^****^ *P*<0.0001 vs. the DMSO group.

To explore the role of OA on SKOV-3 and CAOV3 cells, they were treated with 25, 50, and 100 µg/mL OA for 24 h, respectively. SKOV-3 and CAOV3 cells treated with DMSO were the negative control. The results of the CCK-8 assay showed that there was no significant difference in the cell viability of SKOV-3 and CAOV3 cells between the control and DMSO groups, and OA significantly inhibited the cell viability of SKOV-3 and CAOV3 cells in a dose-dependent manner compared with DMSO (*P*<0.05, **Figure 1A**). Moreover, there were no significant differences in the migration and invasion of SKOV-3 and CAOV3 cells between the control and the DMSO groups, and OA dramatically suppressed cell migration and invasion of SKOV-3 and CAOV3 cells in a dose-dependent way (*P*<0.0001, **Figures 1B–E**). Our data suggested that OA inhibited the cell viability, migration, and invasion of SKOV-3 and CAOV3 cells.

### OA induced autophagy and inhibited glycolysis of SKOV-3 and CAOV3 cells

**Figure 2:**
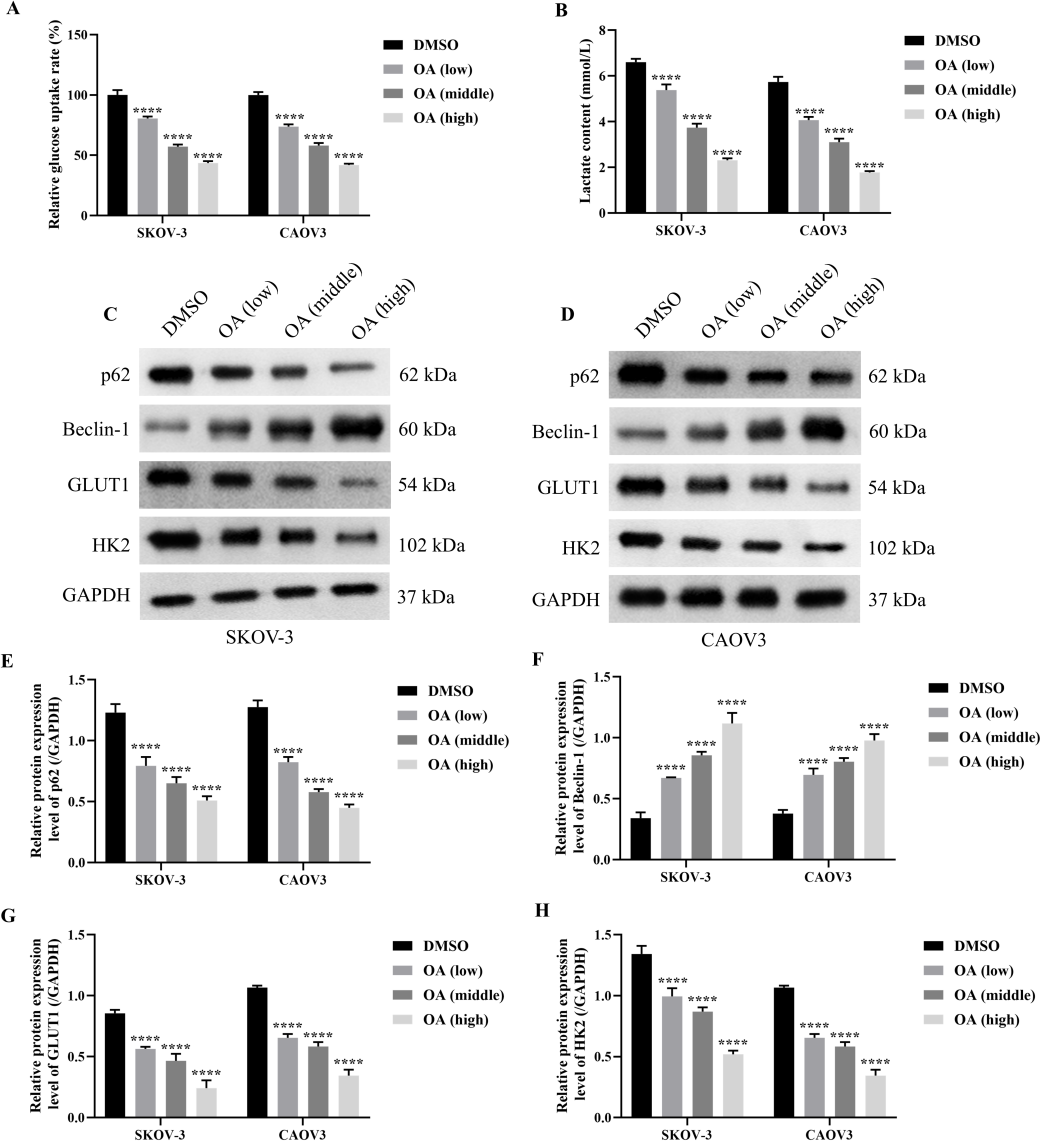
OA induced autophagy and inhibited glycolysis of SKOV-3 and CAOV3 cells. The glucose uptake rate **(A)** and lactate content **(B)** of SKOV-3 and CAOV3 cells were measured using the corresponding kits (n = 3). The protein blots of SKOV-3 **(C)** and CAOV3 **(D)** cells. The protein levels of autophagy markers (p62 **(E)** and Beclin-1 (**F**)) and glycolysis markers (GLUT1 **(G)** and HK2 **(H)** of SKOV-3 and CAOV3 cells were examined using western blotting (n = 3). OA, oleanolic acid; GLUT1, glucose transporter type 1; HK2, hexokinase 2. ^***^ *P*<0.001 and ^****^ *P*<0.0001 vs. the DMSO group.

As the above results have demonstrated the roles of OA on the cell viability, migration, and invasion of SKOV-3 and CAOV3 cells, the effects of OA on the glycolysis and autophagy of SKOV-3 and CAOV3 cells were further investigated. As presented in **Figures 2A, B**, OA significantly reduced the glucose uptake rate and lactate content of SKOV-3 and CAOV3 cells, suggesting that OA might inhibit their glycolysis (*P*<0.001). In addition, the protein levels of autophagy markers (p62 and Beclin-1) and glycolysis markers (GLUT1 and HK2) in SKOV-3 and CAOV3 cells were determined. As illustrated in **Figures 2C–H**, OA dramatically up-regulated Beclin-1 protein expression levels and markedly down-regulated p62, GLUT1, and HK2 protein expression levels. The above results affirmed that OA induced autophagy and suppressed the glycolysis of SKOV-3 and CAOV3 cells.

### Knocking down MiR-122 reversed the effects of OA on the cell viability, migration, and invasion of SKOV-3 and CAOV3 cells

**Figure 3:**
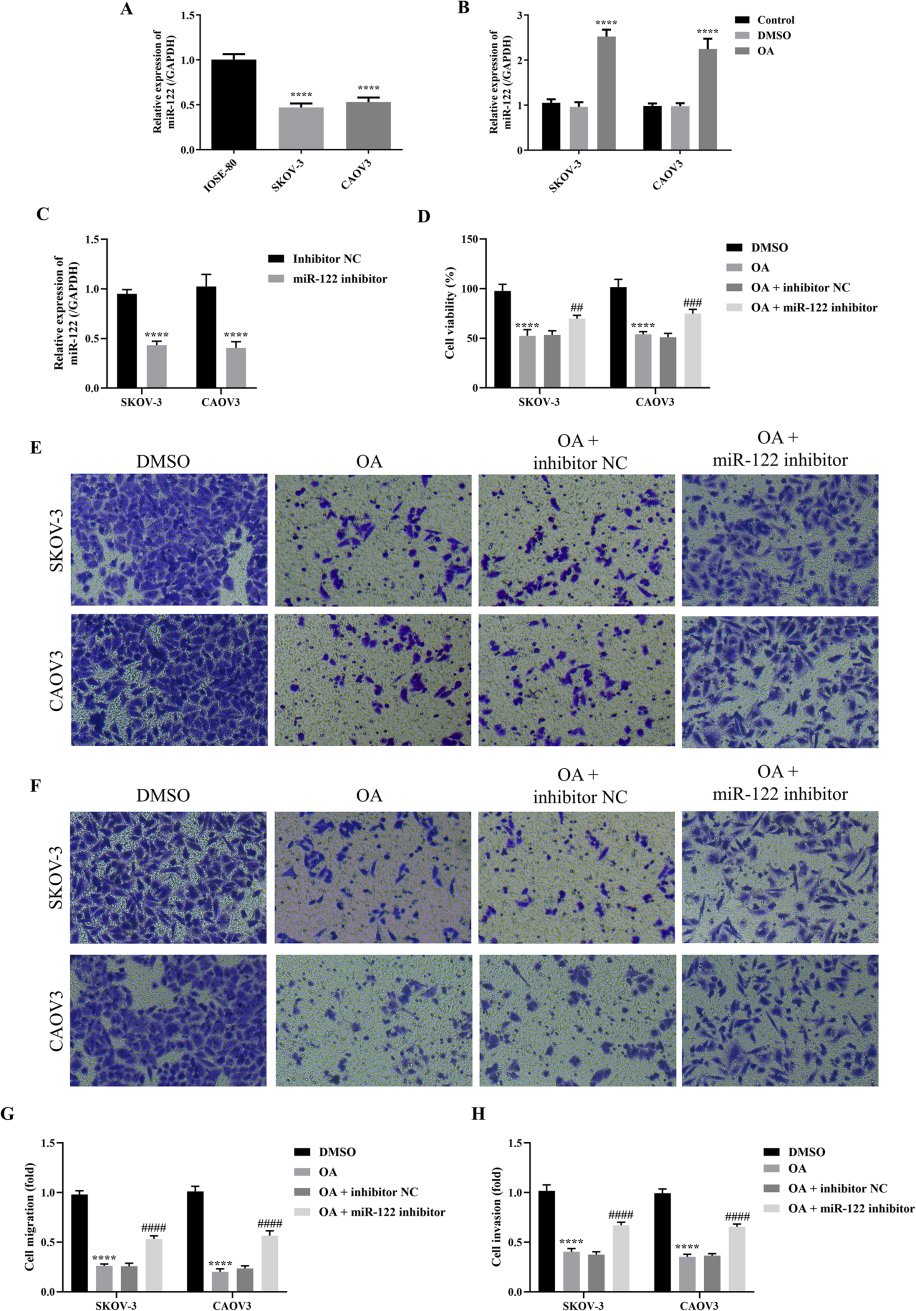
Knocking down miR-122 reversed the effects of OA on the cell viability, migration, and invasion of SKOV-3 and CAOV3 cells. **(A)** The miR-122 expression level in IOSE-80, SKOV-3, and CAOV3 cells (n = 3). **(B)** The miR-122 expression level in SKOV-3 and CAOV3 cells after OA treatment (n = 3). **(C)** The miR-122 expression level in SKOV-3 and CAOV3 cells after knocking down miR-122 (n = 3). **(D)** The cell viability of SKOV-3 and CAOV3 cells was assessed using the CCK-8 approach (n = 3). The representative images of the migrated **(E)** and invaded **(F)** SKOV-3 and CAOV3 cells were detected by the Transwell assay (magnification: 200 ×). The statistical analysis of the cell migration **(G)** and invasion **(H)** of SKOV-3 and CAOV3 cells (n = 5). OA, oleanolic acid. ^**^ *P*<0.01, ^***^ *P*<0.001, and ^****^ *P*<0.0001 vs. the IOSE-80/DMSO/inhibitor NC group; ^#^ *P*<0.05, ^##^ *P*<0.01, and ^####^ *P*<0.0001 vs. the OA + inhibitor NC group.

As it has been revealed that miR-122 suppressed the progression of OC, we subsequently explored the effects of OA on miR-122 expression of SKOV-3 and CAOV3 cells (24, 25). First, the miR-122 expression level between normal ovarian epithelial cells and OC cells was compared. It was shown that the miR-122 expression level in SKOV-3 and CAOV3 cells was significantly down-regulated compared in IOSE-80 cells (*P*<0.0001, **Figure 3A**). Meanwhile, it was found that OA dramatically up-regulated miR-122 expression level in SKOV-3 and CAOV3 cells (*P*<0.0001, **Figure 3B**). Since the above studies revealed that OA inhibited the cell viability, migration, and invasion of SKOV-3 and CAOV3 cells, we subsequently investigated whether knocking down miR-122 reversed the effects of OA on the cell viability, migration, and invasion of SKOV-3 and CAOV3 cells. First, the qRT-PCR approach was used to determine the transfection efficiency. As presented in **Figure 3C**, compared with the inhibitor NC group, the miR-122 expression level of SKOV-3 and CAOV3 cells in the miR-122 inhibitor group was markedly reduced (*P*<0.01), suggesting that the miR-122 inhibitor was successfully transfected. Subsequently, the cell viability, migration, and invasion of SKOV-3 and CAOV3 cells were detected. As illustrated in **Figure 3D**, OA prominently suppressed SKOV-3 and CAOV3 cell viability (*P*<0.0001), while knocking down miR-122 effectively counteracted the effects of OA on the cell viability (*P*<0.05). In addition, the migration and invasion of SKOV-3 and CAOV3 cells were significantly inhibited by treating with OA (*P*<0.0001), and knocking down miR-122 effectively reversed the effects of OA on migration and invasion (*P*<0.0001, **Figures 3E–H**). The above results indicated that knocking down miR-122 effectively reversed the effects of OA on the cell viability, migration, and invasion of SKOV-3 and CAOV3 cells.

### Knocking down MiR-122 reversed the effects of OA on the autophagy and glycolysis of SKOV-3 and CAOV3 cells

**Figure 4:**
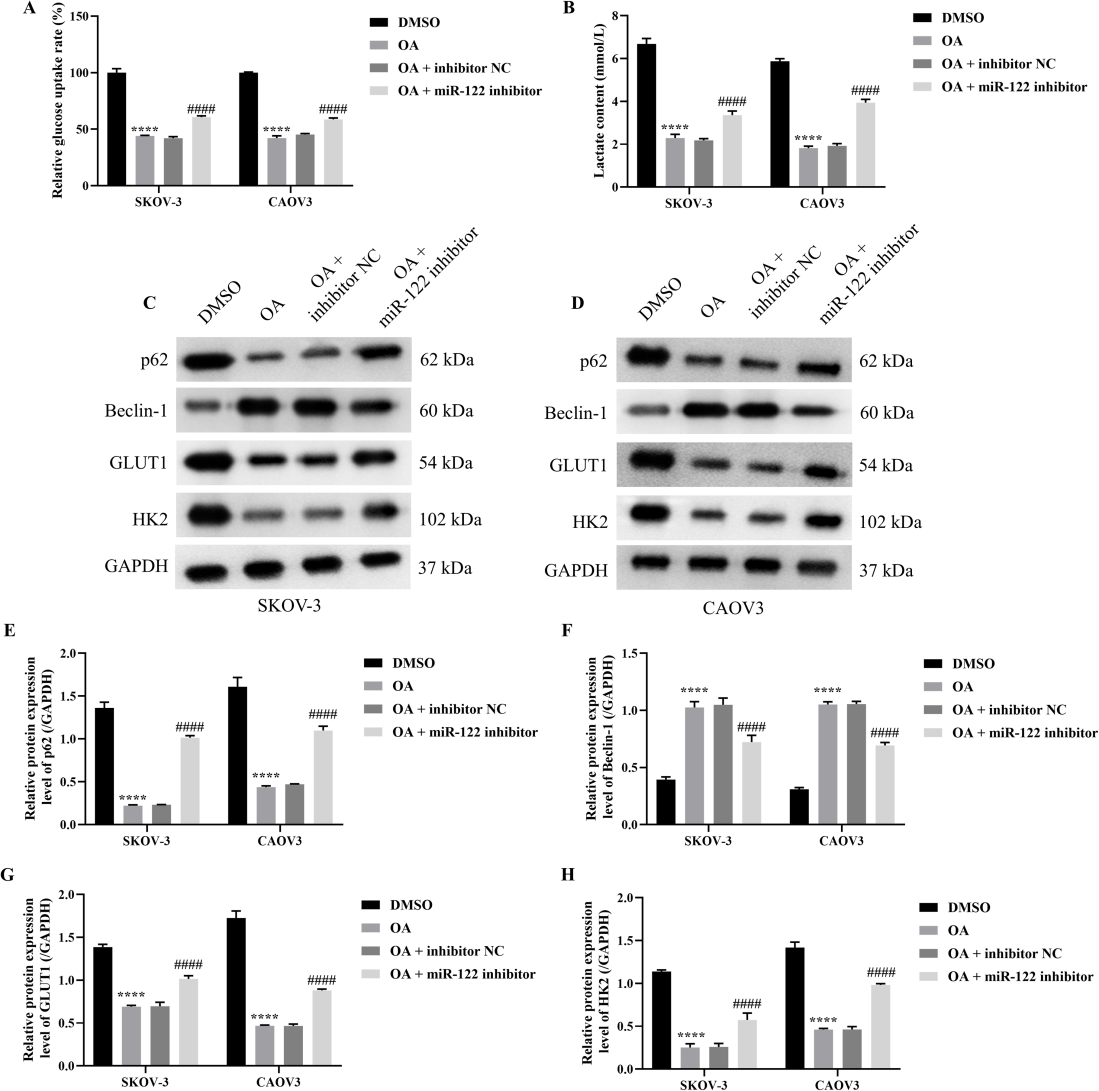
Knocking down miR-122 reversed the effects of OA on autophagy and glycolysis of SKOV-3 and CAOV3 cells. The glucose uptake rate **(A)** and lactate content **(B)** of SKOV-3 and CAOV3 cells were measured using the corresponding kits (n = 3). The protein blots of SKOV-3 **(C)** and CAOV3 **(D)** cells. The protein levels of autophagy markers (p62 **(E)** and Beclin-1 **(F)** and glycolysis markers (GLUT1 **(G)** and HK2 (**H**)) of SKOV-3 and CAOV3 cells were examined using western blotting (n = 3). OA, oleanolic acid; GLUT1, glucose transporter type 1; HK2, hexokinase 2. ^****^ *P*<0.0001 vs. the DMSO group; ^###^ *P*<0.001 and ^####^ *P*<0.0001 vs. the OA + inhibitor NC group.

As the above results demonstrated that OA induced autophagy and inhibited glycolysis of SKOV-3 and CAOV3 cells, and knocking down miR-122 effectively reversed the effects of OA on the cell viability, migration, and invasion of SKOV-3 and CAOV3 cells, we subsequently explored whether knocking down miR-122 reversed the effects of OA on the autophagy and glycolysis of SKOV-3 and CAOV3 cells. As presented in **Figures 4A, B**, OA significantly reduced the glucose uptake rate and lactate content of SKOV-3 and CAOV3 cells (*P*<0.0001), and knocking down miR-122 effectively reversed the effects of OA on the glucose uptake rate and lactate content (*P*<0.001). In addition, as shown in **Figures 4C–H**, OA dramatically up-regulated Beclin-1 protein expression levels (*P*<0.0001) and prominently down-regulated p62, GLUT1, and HK2 protein expression levels (*P*<0.0001). Meanwhile, knocking down miR-122 effectively reversed the effects of OA on p62, Beclin-1, GLUT1, and HK2 protein expression levels (*P*<0.0001). The above results confirmed that knocking down miR-122 reversed the effects of OA on the autophagy and glycolysis of SKOV-3 and CAOV3 cells.

### MiR-122 targeted and inhibited PDK4

**Figure 5:**
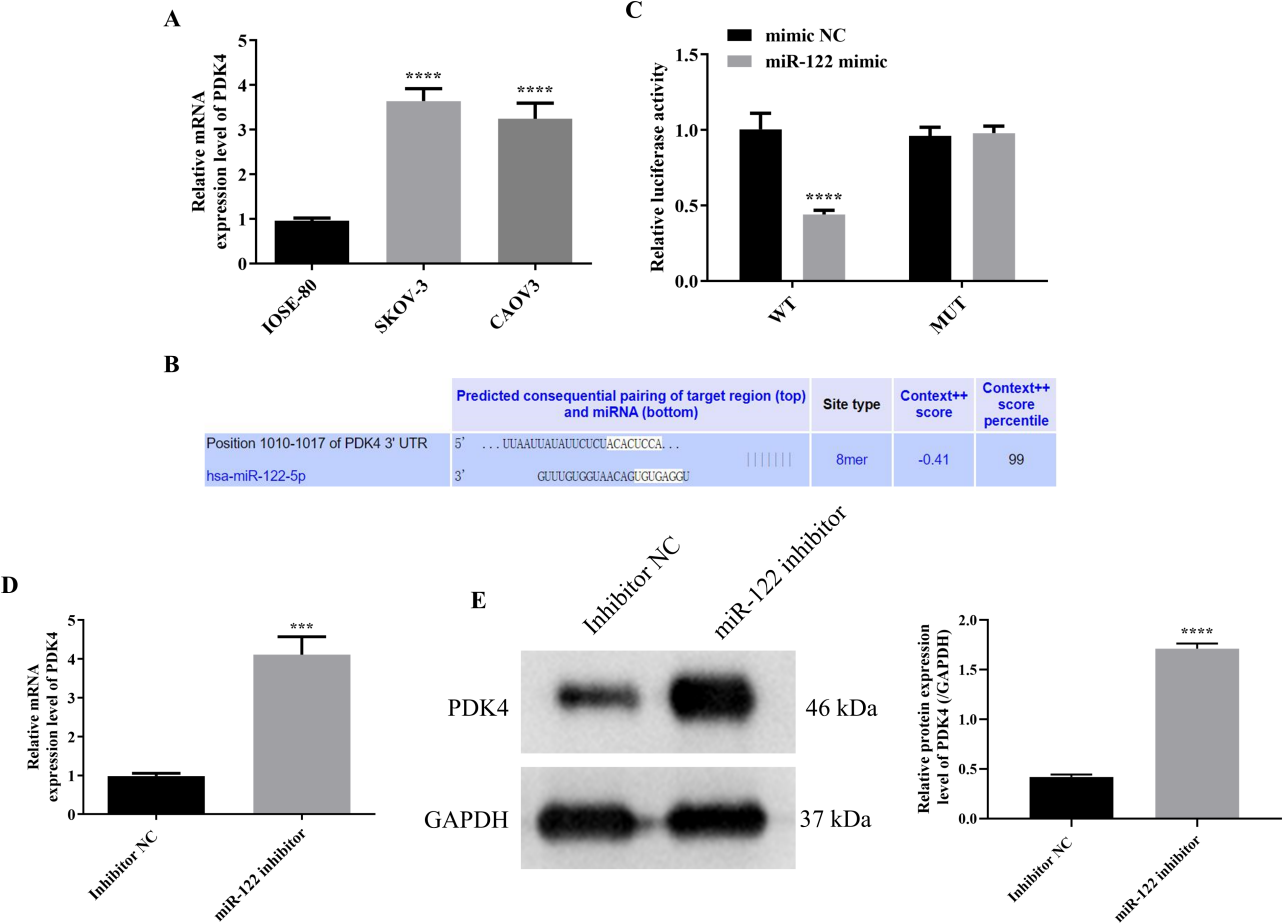
MiR-122 targeted and inhibited PDK4. **(A)** The mRNA protein expression level in IOSE-80, SKOV-3, and CAOV3 cells (n = 3). **(B)** The potential binding sites between miR-122 and PDK4 were predicted using TargetScan database. **(C)** The targeting relationship was confirmed using the luciferase reporter analysis (n = 3). The mRNA **(D)** and protein **(E)** expression levels of PDK4 in SKOV-3 cells after knocking down miR-122 (n = 3). PDK4, pyruvate dehydrogenase kinase 4. ^***^ *P*<0.001 and ^****^ *P*<0.0001 vs. the first group.

Since the previous study demonstrated that overexpressing PDK4 enhanced the cell proliferation, invasion, and chemoresistance in OC, and the above results indicated that knocking down miR-122 up-regulated the cell viability, migration, and invasion of SKOV-3 and CAOV3 cells, we speculated that PDK4 might be a downstream target of miR-122 (26). Therefore, after determining that PDK4 was significantly up-regulated in SKOV-3 and CAOV3 cells compared to IOSE-80 cells (*P*<0.0001, **Figure 5A**), we subsequently explored the targeting relationship between miR-122 and PDK4. Due to the similar results in SKOV-3 and CAOV3 cells, we randomly chose one cell line (SKOV-3 cells) for the following study. After predicting the binding sites between miR-122 and PDK4 with the help of the TargetScan online database (**Figure 5B**), the dual luciferase reporter analysis was performed to determine the targeted relationship between miR-122 and PDK4. As presented in **Figure 5C**, although there were no significant differences in the luciferase activity between the mimic NC and miR-122 mimic groups in SKOV-3 cells transfected with MUT-PDK4, the luciferase activity of SKOV-3 cells transfected with WT-PDK4 in the miR-122 mimic group was significantly lower than that in the mimic NC group, indicating that PDK4 was the downstream target of miR-122 (*P*<0.0001). In addition, the mRNA and protein expression levels of PDK4 in SKOV-3 cells were prominently down-regulated after knocking down miR-122 (*P*<0.001, **Figures 5D, E**). The above results affirmed that miR-122 targeted and inhibited PDK4.

### OA induced autophagy and inhibited glycolysis of SKOV-3 cells via regulating the MiR-122/PDK4 axis

**Figure 6:**
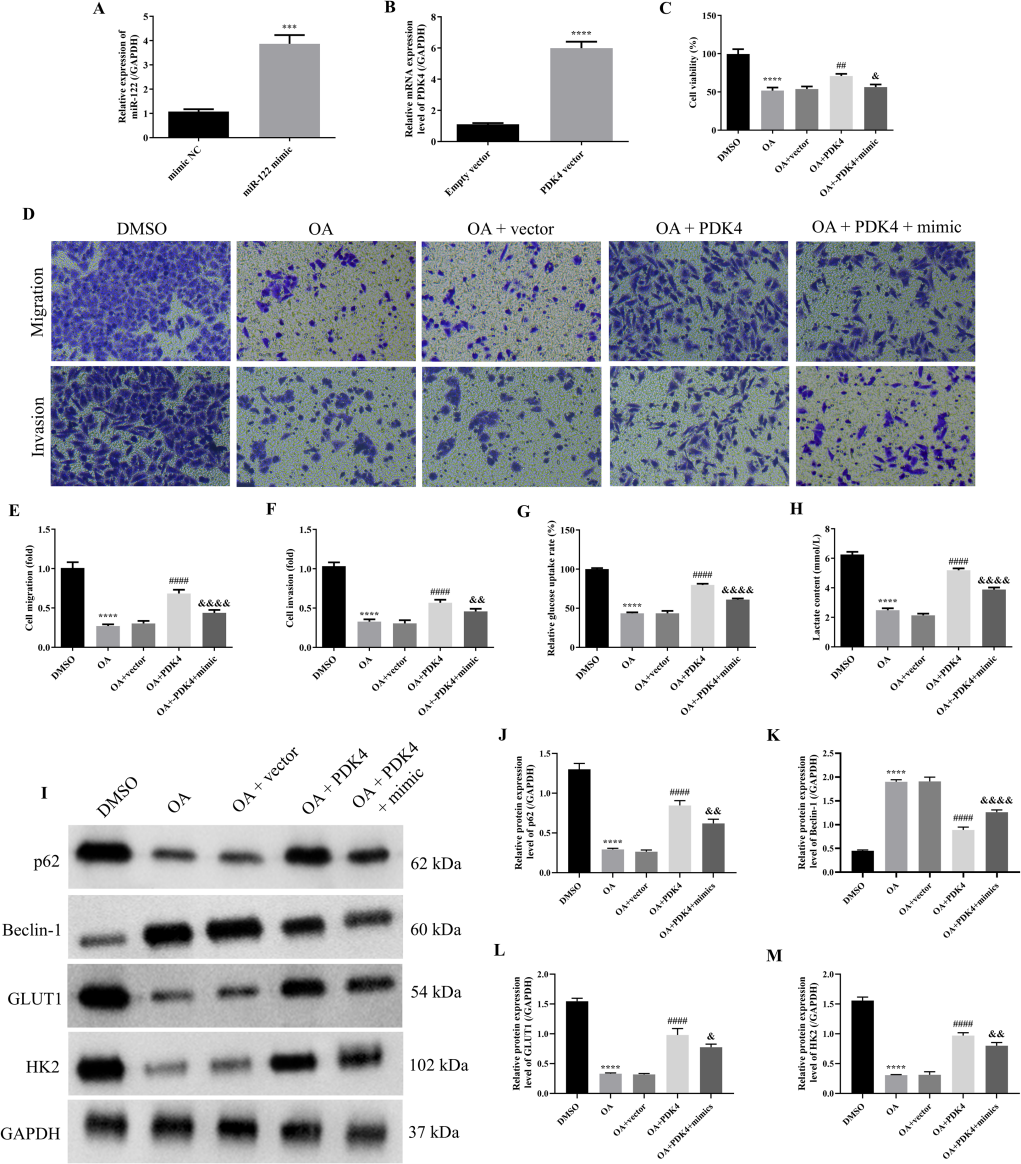
OA induced autophagy and inhibited glycolysis of SKOV-3 cells via regulating the miR-122/PDK4 axis. The miR-122 expression level **(A)** and PDK4 mRNA expression level **(B)** of SKOV-3 cells (n = 3). **(C)** The cell viability of SKOV-3 cells was determined by the CCK-8 approach (n = 3). **(D)** The representative images of the migrated and invaded SKOV-3 and CAOV3 cells were detected by the Transwell assay (magnification: 200 ×). The statistical analysis of the cell migration **(E)** and invasion **(F)** of SKOV-3 and CAOV3 cells (n = 5). The glucose uptake rate **(G)** and lactate content **(H)** of SKOV-3 cells were assessed using corresponding kits (n = 3). **(I)** The protein blots of SKOV-3 cells. The protein levels of autophagy markers (p62 **(J)** and Beclin-1 **(K)**) and glycolysis markers (GLUT1 **(L)** and HK2 **(M)**) of SKOV-3 cells were examined using western blotting (n = 3). OA, oleanolic acid; GLUT1, glucose transporter type 1; HK2, hexokinase 2; PDK4, pyruvate dehydrogenase kinase 4. ^***^ *P*<0.001 and ^****^ *P*<0.0001 vs. the first group; ^##^ *P*<0.01 and ^####^ *P*<0.0001 vs. the OA + vector group; ^&^ *P*<0.05, ^&&^ *P*<0.01, and ^&&&&^ *P*<0.0001 vs. the OA + PDK4 group.

As the above results proved that miR-122 targeted and inhibited PDK4, and knocking down miR-122 effectively reversed the effects of OA on the cell viability, migration, invasion, autophagy, and glycolysis of SKOV-3 and CAOV3 cells, the rescue experiments were performed to explore whether OA inhibited the biological behaviors of SKOV-3 cells via the miR-122/PDK4 axis. First, the qRT-PCR approach was used to investigate whether the miR-122 mimic and PDK4 vector were successfully transfected. As illustrated in **Figures 6A, B**, compared with the mimic NC and empty vector groups, the miR-122 and PDK4 mRNA expression levels of SKOV-3 cells in the miR-122 mimic and PDK4 vector groups were significantly up-regulated (*P*<0.0001), suggesting that the miR-122 mimic and PDK4 vector were successfully transfected into SKOV-3 cells. Subsequently, the cell viability, migration, and invasion of SKOV-3 cells were determined. As presented in **Figures 6C–F**, compared with the DMSO group, the cell viability, migration, and invasion of SKOV-3 cells were significantly reduced (*P*<0.0001). Meanwhile, the additional overexpression of PDK4 effectively reversed the effects of OA on the cell viability, migration, and invasion of SKOV-3 cells (*P*<0.01). In addition, the additional overexpression of miR-122 prominently reversed the effects of overexpressing PDK4 on the cell viability, migration, and invasion of SKOV-3 cells (*P*<0.05). After that, we further explored whether OA induced autophagy and inhibited glycolysis of SKOV-3 cells via the miR-122/PDK4 axis. As shown in **Figures 6G, H**, OA dramatically decreased the glucose uptake rate and lactate content (*P*<0.0001). Overexpressing PDK4 effectively reversed the effects of OA on the glucose uptake rate and lactate content of SKOV-3 cells (*P*<0.0001). Meanwhile, the overexpression of miR-122 also effectively reversed the effects of overexpressing PDK4 on these indicators of SKOV-3 cells (*P*<0.0001). In addition, as illustrated in **Figures 6I–M**, OA markedly down-regulated the protein expression levels of p62, GLUT1, and HK2 but up-regulated the Beclin-1 protein expression level of SKOV-3 cells (*P*<0.0001). Overexpressing PDK4 effectively reversed the effects of OA on the protein expression levels of p62, Beclin-1, GLUT1, and HK2 of SKOV-3 cells (*P*<0.0001). Additionally, the overexpression of miR-122 effectively reversed the effects of overexpressing PDK4 on these protein expression levels of SKOV-3 cells (*P*<0.05). The above results demonstrated that OA induced autophagy and inhibited glycolysis of SKOV-3 cells via the miR-122/PDK4 axis.

### OA induced autophagy and inhibited glycolysis of OC via regulating the MiR-122/PDK4 axis *in vivo*

**Figure 7:**
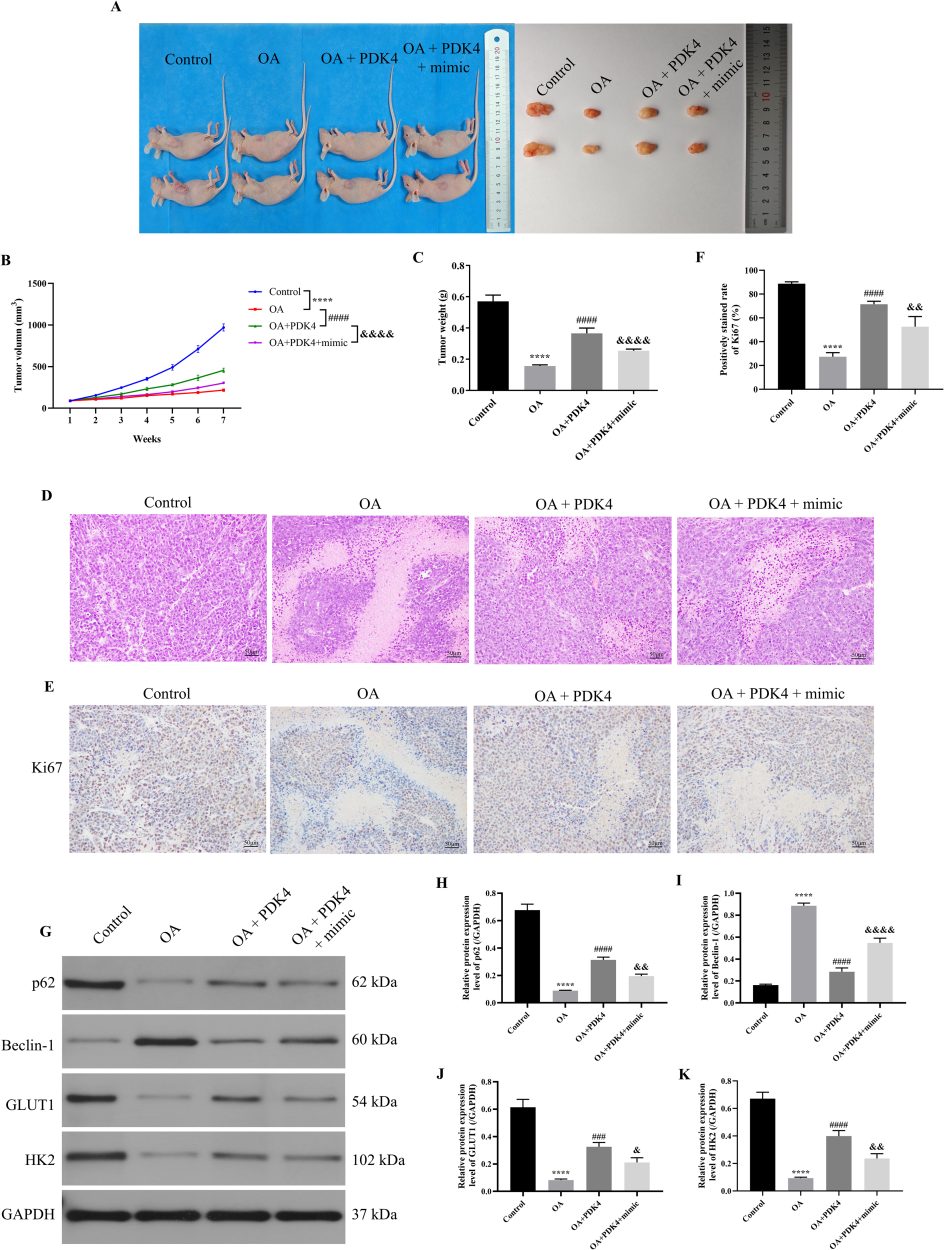
OA induced autophagy and inhibited glycolysis of OC via regulating the miR-122/PDK4 axis *in vivo*. **(A)** The representative images of the tumor in each group. **(B)** The tumor volume was calculated weekly (n = 6). **(C)** The tumor weight was measured (n = 6). **(D)** The pathological state of tumors was analyzed by H&E staining. **(E)** The representative IHC staining results of the tumor. **(F)** The Ki67 expression in the tumor was analyzed (n = 3). **(G)** The protein blots of tumor tissue. The protein levels of autophagy markers (p62 **(H)** and Beclin-1 **(I)**) and glycolysis markers (GLUT1 **(J)** and HK2 **(K)**) of tumor tissue were examined using western blotting (n = 3). OA, oleanolic acid; OC, ovarian cancer; IHC, immunohistochemical; GLUT1, glucose transporter type 1; HK2, hexokinase 2. ^****^ *P*<0.0001 vs. the control group; ^###^ *P*<0.001 and ^####^ *P*<0.0001 vs. the OA group; ^&^ *P*<0.05, ^&&^ *P*<0.01, and ^&&&&^ *P*<0.0001 vs. the OA + PDK4 group.

As the above results indicated that OA induced autophagy and inhibited glycolysis of SKOV-3 cells via the miR-122/PDK4 axis, we further explored whether OA induced autophagy and inhibited glycolysis of OC via the miR-122/PDK4 axis *in vivo*. As presented in **Figures 7A–C**, compared with the control group, the tumor volume and weight were significantly reduced (*P*<0.0001). Overexpressing PDK4 effectively reversed the effects of OA on the volume and weight of tumor tissue (*P*<0.0001). The overexpression of miR-122 effectively reversed the effects of overexpressing PDK4 on these indicators of the tumor tissue (*P*<0.0001). In addition, as shown in **Figure 7D**, in the control group, the number of tumor cells was large, the tumor cells were tightly arranged, and their nucleus were deeply stained. Meanwhile, after being treated with OA, the number of tumor cells was reduced, the tumor cells were sparsely arranged, and their nucleus were shallowly stained. The overexpression of PDK4 and miR-122 effectively reversed the effects of OA and the overexpression of PDK4 on the above alterations in the tumor tissue, respectively. Moreover, OA also dramatically reduced Ki67 expression in the tumor tissue (*P*<0.0001, **Figures 7E, F**). The overexpression of PDK4 and miR-122 effectively reversed the effects of OA and the overexpression of PDK4 on the Ki67 expression in the tumor tissue, respectively (*P*<0.01). Then, the protein expression levels of the autophagy markers, p62 and Beclin-1, and glycolysis markers, GLUT1 and HK2, in tumor tissue were determined. As illustrated in **Figures 7G–K**, after administering OA, the protein expression level of Beclin-1 was prominently up-regulated, and the protein expression levels of p62, GLUT1, and HK2 were markedly down-regulated in the tumor tissue (*P*<0.0001). The overexpression of PDK4 and miR-122 effectively reversed the effects of OA and overexpressing PDK4 on the protein expression levels of Beclin-1, p62, GLUT1, and HK2 in the tumor tissue, respectively (*P*<0.05). The above results confirmed that OA induced autophagy and inhibited glycolysis of OC via the miR-122/PDK4 axis *in vivo*.

## Discussion

Although several preclinical findings have shown that EHT5372 and R05454948 produced effects in OC cell lines, more effective treatments are required due to the prevalence of drug resistance *in vivo* (27). The study of natural products in diseases is increasing. OA cannot be used directly for disease treatment due to its water insolubility, but a variety of OA derivatives can be used to alleviate or treat disease. Therefore, elucidating the mechanism of OA is important for its derivatives to play a clinical role (28). OA can decelerate the progression of numerous diseases, such as Alzheimer’s disease, diabetes mellitus, inflammation, and cancer (29–31). Previous studies have revealed that OA suppressed OC development by regulating multiple biological functions, including cellular proliferation, metastasis, and apoptosis (17, 32). In this study, we observed that OA suppressed the cell viability, migration, and invasion of OC cells *in vitro* and the tumor growth and Ki67 expression of the tumor tissue *in vivo*, and promoted the pathological damages of tumor tissue *in vivo*, which were consistent with previous studies mentioned above.

Tumor development, metastasis and drug resistance are still major problems in the clinical treatment of OC. Autophagy has the function to promote or inhibit OC development. In detail, tumor cells have drug resistance to chemoradiotherapy by utilizing autophagy. On the other hand, autophagy enhances drug cytotoxicity to induce tumor cell death (33). Additionally, glycolysis epigenetic changes in tumor cells and thereby promoting OC growth, metastasis, and drug resistance (34). Glycolysis drives tumor progression by regulating autophagy capability, and autophagy participates in energy metabolism, including glycolysis (35, 36). The previous studies demonstrated that OA induced autophagy in colon cancer cells by regulating the p38/FOXO3a/Sirt6 pathway, and in hepatoma cells through the Akt/mTOR pathway, reflected in the decreased p62 expression and increased LC3-II and Beclin-1 expression (37, 38). In addition, the previous studies also suggested that OA inhibited the glycolysis of prostate carcinoma, breast cancer, and gastric cancer (19, 39). However, it is still unclear whether OA influences the autophagy and glycolysis of OC. Therefore, after determining the effects of OA on OC cells, the effects of OA on the autophagy and glycolysis of OC were further investigated. In the present study, we demonstrated that OA induced autophagy and inhibited glycolysis of OC *in vivo* and *in vitro*, manifested as the up-regulation of Beclin-1 protein expression level and the down-regulation of protein expression levels of p62, GLUT1, and HK2, which were consistent with previous studies mentioned above. It is proven that inhibiting glycolysis blunts hypoxia-inducible factor-1α (HIF-1α) accumulation (40). The suppressed HIF-1α prevented the OC progression by down-regulating angiogenic factors to reduce angiogenesis, inhibiting matrix metalloproteinases and key transcription factors for epithelial-mesenchymal transition to reduce the metastasis, and improving the immune microenvironment to promote the recovery of T cell killing function (41–43).

MiRNAs are crucial in physiological and pathological processes by modulating cell growth, differentiation, immune response, angiogenesis, and tumorigenesis (44). OA functions in disease by regulating the expression of multiple miRNAs. For example, OA inhibits Hippocampal neuronal cell pyroptosis by downregulating miR-186-5p (45). OA impedes dysfunctions of chondrocytes by regulating miR-148-3p, which targets FGF2 expression (46). In addition, OA promoted Treg/Th17 balance to inhibit gastric cancer by increasing miR-98-5p expression (47). It was reported that OA also suppressed the migration and epithelial-mesenchymal transition of hepatocellular carcinoma cells by inhibiting miR-130b-3p-induced macrophage M2 polarization and glycolysis (48). Moreover, the previous studies also demonstrated that OA suppressed lung cancer and liver cancer development by elevating the levels of miR-122 (49, 50). However, whether OA can affect OC by regulating miR-122 remains unknown. In the study, we discovered that OA up-regulated miR-122 expression level in OC cells. In addition, we also found that knocking down miR-122 abrogated OA-induced inhibition of cell viability, migration, invasion, and glycolysis and the promotion of autophagy of OC cells, indicating that OA might decelerate the progression of OC by up-regulating miR-122 expression.

PDK4 is a mitochondrial matrix enzyme that regulates energy metabolism (51). It was reported that PDK4 facilitates tumor cell proliferation, invasion, glycolysis, and chemotherapy resistance in OC (26, 52). In the regulation of glycolysis of tumor cells, depletion of PDK4 reduces glucose consumption, lactic acid, and ATP production (53). In addition, it was demonstrated that OA improved glucose consumption and lactate production to modulate the energy metabolism of breast cancer by regulating the key rate-limiting enzymes of aerobic glycolysis (54). However, it is still unclear whether OA targeted regulates PDK4 in OC. Therefore, we first investigated whether regulating PDK4 affects the effects of OA on OC. Our studies found that overexpressing PDK4 counteracted the suppression of cell viability, migration, invasion, and glycolysis and the promotion of autophagy of OC cells induced by OA *in vitro*. Meanwhile, overexpressing PDK4 reversed the inhibition of tumor volume and weight, Ki67 expression, and glycolysis, and the promotion of pathological injuries and autophagy of tumor tissue induced by OA *in vivo*. As the above studies demonstrated that inhibiting miR-122 also reversed the effects of OA on OC, we speculated that there might be a targeted relationship between miR-122 and PDK4. In the present study, we identified that PDK4 is a miR-122 target with the help of dual luciferase reporter analysis as well as qRT-PCR and Western blotting approaches. In addition, one previous study also affirmed that miR-122-5p suppresses neuropathic pain development by targeting PDK4, which proves our findings (55). Thus, we further evaluated whether OA functioned through the miR-122/PDK4 axis. The results showed that, *in vivo* and *in vitro*, overexpressing PDK4 effectively reversed the effects of OA on the autophagy and glycolysis of OC, and overexpressing miR-122 produced the opposite effects to overexpressing PDK4, suggesting that OA induced autophagy and inhibited glycolysis to attenuate OC progression by regulating the miR-122/PDK4 axis.

**Figure 8:**
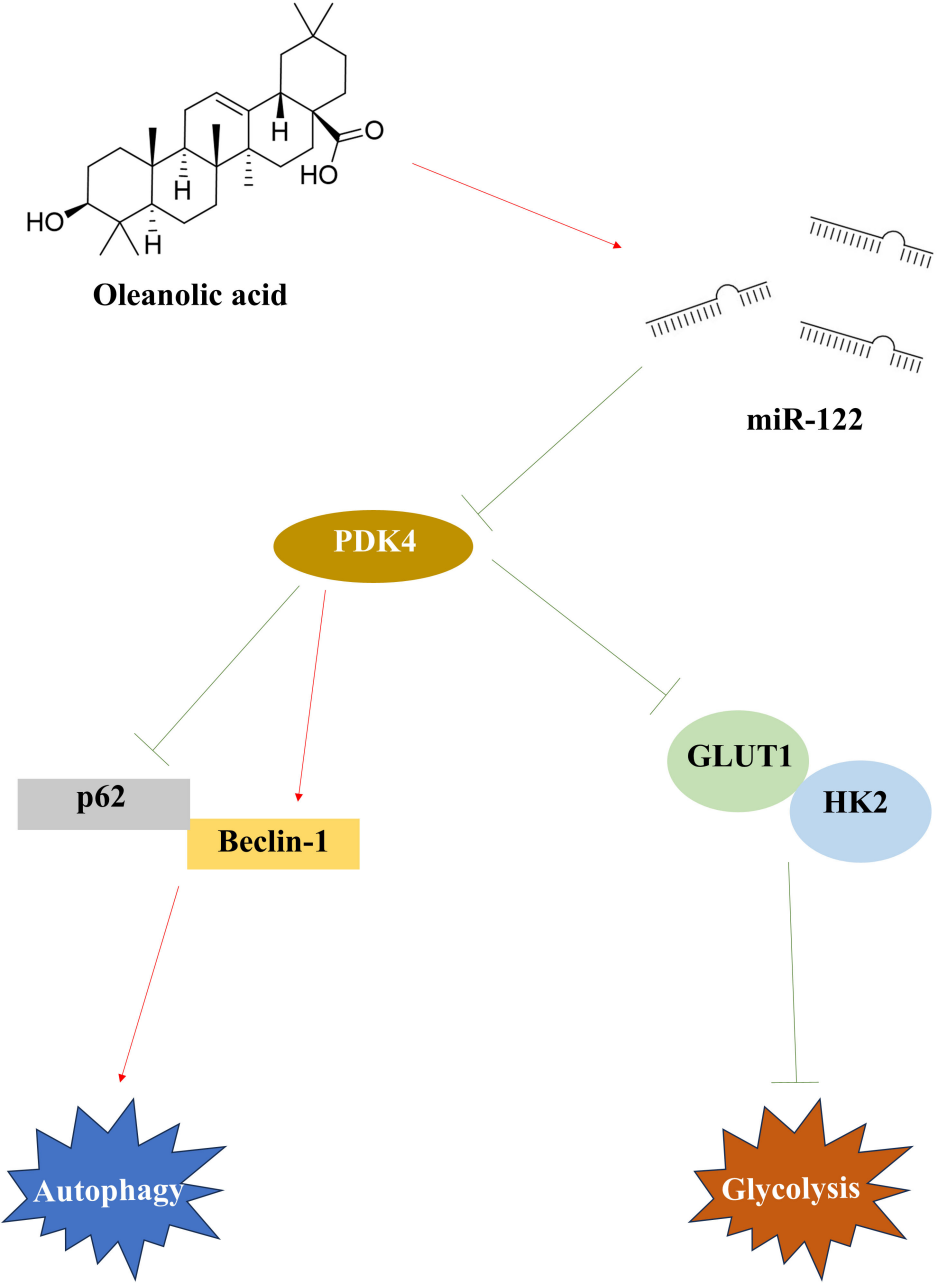
The therapeutic mechanisms of OA on OC. After being treated with OA, miR-122 was up-regulated and PDK4 was down-regulated in OC. In OC, the down-regulated PDK4 induced autophagy by promoting Beclin-1 expression and reducing p62 expression, and inhibited glycolysis by suppressing the GLUT1 and HK2 expression. OA, oleanolic acid; OC, ovarian cancer; PDK4, pyruvate dehydrogenase kinase 4; GLUT1, glucose transporter type 1; HK2, hexokinase 2.

Taken together, OA inhibited the cell viability, migration, and invasion of OC cells *in vitro*, suppressed the tumor volume and weight and Ki67 expression, and induced pathological injuries of tumor tissue *in vivo*. In addition, knocking down miR-122 effectively reversed the effects of OA on OC cells mentioned above, and overexpressing PDK4 effectively reversed the effects of OA on OC cells and tumor tissue mentioned above. Moreover, overexpressing miR-122 produced the opposite effects to overexpressing PDK4. In conclusion, OA induced autophagy and inhibited glycolysis to attenuate OC progression by regulating the miR-122/PDK4 axis (**Figure 8**), providing a theoretical basis for clinical treatment of OC with OA and novel therapeutic targets of OC. However, there are still some shortcomings in this study. Although our studies demonstrated that OA effectively induced autophagy and inhibited glycolysis to attenuate OC progression, it is still unclear whether there is a crosstalk between autophagy and glycolysis. The current studies have indicated that the cross-talk between autophagy and glycolysis regulates senescence and stemness of tumor subpopulations, and lactate was a bridge linking glycolysis and autophagy through lactylation (56, 57). As our studies have demonstrated that OA significantly reduced the lactate content in SKOV-3 and CAOV3 cells, we consider that the effects of OA on the cross-talk between autophagy and glycolysis in OC should be further investigated. In addition, although the targeted relationship between OA and miR-122 and between miR-122 and PDK4 has been affirmed in our studies, there are still some other possible pathways acting. The previous studies have reported that OA alleviated gastric cancer by up-regulating miR-98-5p expression, and miR-98-5p also played an essential role in the progression of OC (47, 58). In addition, the previous studies also demonstrated that P4HA1 and SLC1A5 acted as the downstream targets of miR-122 in OC (24, 25). Therefore, we consider that the gene sequencing technologies could be used to comprehensively discover the differentially expressed genes in OC after being treated with OA in subsequent studies, providing a more comprehensive theoretical basis for the clinical treatment of OA in OC. Moreover, although our studies have affirmed that OA induced autophagy and inhibited glycolysis to attenuate OC progression by regulating the miR-122/PDK4 axis, it is still unclear whether OA also affects the immune cells in the tumor microenvironment of OC. The previous studies demonstrated that OA not only regulated the Treg/Th17 imbalance but also down-regulated PD-L1 in gastric cancer (47, 59). Therefore, we believe that in the future, the effects of OA on the immune cells in the tumor microenvironment of OC should be further investigated to broaden the implications for clinical application.

## Data Availability

The raw data supporting the conclusions of this article will be made available by the authors, without undue reservation.
